# Breast self-examination, mammography and Pap test among Turkish women: Role of healthcare professionals in Sivas

**DOI:** 10.18332/ejm/124112

**Published:** 2020-08-04

**Authors:** Z. Burcu Yurtsal, Mine Bekar, Handan Güler, Perihan Çetin, Büşra Cesur

**Affiliations:** 1 Department of Midwifery,Faculty of Health Sciences, Sivas Cumhuriyet University, Sivas,Turkey; 2 Patient Care Service, Izmir Katip Celebi University, Izmir,Turkey

**Keywords:** breast self-examination, mammography, Pap test, Turkish women, midwives

## Abstract

**INTRODUCTION:**

The aim of the present study is to determine how often women perform breast self-examination (BSE) and undergo mammography and Pap test, and how healthcare professionals affect them to do so.

**METHODS:**

This descriptive study was carried out with 1025 women, aged 20–82 years and living in the central district of Sivas, who presented to the Gynecology and Obstetrics Outpatient Clinics of Hospitals between January and December 2010, and who volunteered to participate in the study and gave their verbal agreement to the researchers.

**RESULTS:**

Out of the participants, 46.9% lacked mammogram knowledge, 13.2% received advice from healthcare professionals, and 20.1% thought that they were healthy and thus did not need mammography. Out of the participants, 45.9% lacked knowledge about the Pap test, 11.8% received advice from healthcare professionals, and 18.9% thought that they were healthy and thus did not need a ‘Pap test’.

**CONCLUSIONS:**

While one-fourth of the participants stated that they performed BSE and underwent mammography at least once, more than half reported that they did not have a Pap test. In preventive healthcare services, periodic health examinations and screenings for the most common types of women cancers should be increased; midwives should give education and counseling, and the importance of practices aimed at raising social awareness should be emphasized.

## INTRODUCTION

According to the World Health Organization (WHO), while 16.1 million women were diagnosed with breast cancer in 2012, about 0.53 million women were diagnosed with cervical cancer^1^. In Turkey, of the 10 most common types of women cancers, breast cancer ranks first (41.8/100000), while cervical cancer ranks tenth (4.3/100000)^2^. The incidence of breast cancer has been on the increase for a long time in both developed and developing countries^3^. It is the leading cause of cancer-related deaths in women and accounts for 16% of cancer deaths in adult women^4^. If the breast cancerrelated morbidity and mortality are to be reduced, it is of great importance to inform people about the signs and symptoms of breast cancer and to encourage them to perform breast self-examination (BSE) or to have a clinical breast examination (CBE) or mammogram for the early detection of breast cancer. To achieve this goal, the target population’s culture should be taken into account, because this will encourage them to present to hospitals more^5^. Here, the primary responsibility lies with healthcare professionals, particularly with those who regularly meet or communicate with women^6^.

With about 0.5 million new cases, cervical cancer is the second most common type of cancer among women throughout the world and is responsible for 0.25 million deaths each year^7^. Pap smear, also called Pap test, is recommended as a screening method for cervical cancer^8^. In Turkey, screening studies including mammography and Pap test, are performed in the Cancer Early Diagnosis, Screening and Training Centers which are structured by the Cancer Control Department of the Ministry of Health^9^. The most important factor affecting the prognosis in breast and cervical cancer is early diagnosis. Owing to mammography screening, it has been possible to make an early diagnosis, which is reported to reduce mortality by up to 30%^10^. In developed countries, the incidence of cervical cancer has significantly decreased with the introduction of Pap test screening^11^.

The aim of this short report is to determine how often women perform breast self-examination and undergo mammography and Pap test, and how healthcare professionals affect them to do so.

## METHODS

This descriptive study was carried out with 1025 women aged 20–82 years and living in the central district of Sivas. They presented to the Gynecology and Obstetrics Outpatient Clinics of Hospitals between January and December 2010, volunteered to participate in the study, and gave their verbal agreement to the researchers. The study data were collected using the ‘Breast Self-Examination, Mammography and Pap Smear Information Form’ developed by the researchers based on the pertinent literature. The form was filled in by the researchers through face-to-face interviews. Frequencies and the results of the paired t-test and chi-squared test were interpreted using the SPSS version 14.0, and p-values less than 0.05 were considered statistically significant.

## RESULTS

The mean age of the participants in the present study was 51 years (range: 20–82). In all, 50.6% were primary school graduates, 94.2% were homemakers, 90.0% had health insurance, 86.3% were married, and their mean age at marriage was 18 years. Their monthly household income on average was 843 TRY (about 547 US$ according to the exchange rate in 2010).

Some characteristics of the participants regarding performing BSE, displaying screening mammography behaviour and influencing factors are shown in Table 1. Of the participants, 25.6% had performed BSE, 34.5% had undergone mammography, while 68.8% and 63.7% obtained advice from a midwife on BSE and mammography, respectively. Barriers to screening mammography are shown in Table 2. In all, 46.9% lacked mammogram knowledge, 13.2% received advice from healthcare professionals, and 20.1% thought that they were healthy and thus did not need mammography.

**Table 1:** Performing BSE, displaying screening mammography behavior and influencing factors (N=1025), Sivas, Turkey, 2010

*Variables*	*n*	*%*
**Having performed BSE**		
Yes	262	25.6
No	763	74.4
**Having undergone mammography**		
Yes	353	34.5
No	672	65.5
**Planning to undergo mammography (within next 1–5 years)**		
Yes	417	40.7
No	608	59.3
**BSE explained**		
Yes	279	27.2
No	746	72.8
**Those who gave advice on BSE (n=522)**		
Midwife	359	68.8
Nurse	100	19.2
Physician	63	12.0
**Mammogram explained**		
Yes	175	17.1
No	850	82.9
**Those who gave advice on mammography (n=430)**		
Midwife	274	63.7
Nurse	68	15.8
Physician	88	20.5
**Total**	1025	100.0

**Table 2:** Barriers to screening mammography (N=1025)

*Variables*	*n*	*%*
**Lack of mammogram knowledge**		
Yes	481	46.9
No	544	53.1
**Receiving advice from healthcare professionals**		
Yes	135	13.2
No	890	86.8
**Perceiving mammography as a painful and unbearable procedure**		
Yes	54	5.3
No	971	94.7
**Thinking that they are healthy, thus did not need mammography**		
Yes	206	20.1
No	819	79.9
**Total**	1025	100.0

Some characteristics of the participants regarding behaviour towards Pap test screening and influencing factors are given in Table 3. Of the participants, 38.5% had undergone a Pap test, 38.7% planned to undergo a Pap test (within the next 1–5 years), and 70.2% received advice from a midwife. Barriers to screening Pap test are noted in Table 4. Of the participants, 45.9% lacked knowledge about the Pap test, 11.8% received advice from healthcare professionals, 18.9% thought that they were healthy and thus did not need a ‘Pap test’.

**Table 3:** Displaying screening Pap test behavior and influencing factors (N=1025), Sivas, Turkey

*Variables*	*n*	*%*
**Having undergone a Pap test**		
Yes	395	38.5
No	630	61.5
**Planning to undergo a Pap test (within next 1–5 years)**		
Yes	397	38.7
No	628	61.3
**Pap test explained**		
Yes	152	14.8
No	873	85.2
**Those who gave advice on Pap test (n=410)**		
Midwife	288	70.2
Nurse	32	7.8
Physician	90	22.0
**Total**	1025	100.0

**Table 4:** Barriers to Pap test screening (N=1025)

*Variables*	*n*	*%*
**Lack of Pap test knowledge**		
Yes	470	45.9
No	555	54.1
**Receiving advice from healthcare professionals**		
Yes	121	11.8
No	904	88.2
**Perceiving Pap smear as a painful and unbearable procedure**		
Yes	46	4.5
No	979	95.5
**Thinking that they are healthy, thus did not need a ‘Pap test’**		
Yes	194	18.9
No	831	81.1
**Total**	1025	100.0

## DISCUSSION

There has been significant improvement in breast cancer outcomes owing to BSE and mammography screening, because they have contributed to the early detection/early diagnosis of breast cancer^12^. As stated in the literature, among barriers perceived by women preventing them from participating in early diagnosis and screening practices of breast and cervical cancer are fear of cancer, lack of transportation to health facilities, lack of knowledge about cancer and early diagnosis^13,14^. In the present study, of the participating women, 27.2% were informed about BSE, 68.8% were advised to perform BSE by midwives, 17.1% were informed about mammography and 46.9% lacked knowledge about mammography, which is consistent with the results in the literature indicating that women still lack knowledge about these issues.

Although screening methods help to reduce breast and cervical cancer-related mortality, studies conducted on the issues indicate that women do not have sufficient knowledge of screening methods or that they avoid using these methods^15,16^. These studies also indicate that women’s awareness of cancer is not adequate and they have negative judgments and beliefs about screening methods^15,16^.

According to the literature, owing to screening methods such as BSE and mammography, breast cancer can be diagnosed at an early stage and thus treated^15,17^. However, studies conducted on the issue indicate that women are not knowledgeable enough about screening methods or they do not perform BSE and do not undergo mammography regularly, even if they are knowledgeable enough^18,19^. In Turkey, studies conducted with women, whose characteristics vary, draw attention to the fact that the BSE rate is low^15,17^. In their study, Altunkan et al.^20^ determined that none of the women between the ages of 20 and 60 years performed BSE regularly. In the Duman et al.^15^ study, while only 10% of the women performed BSE but occasionally, only two women underwent mammography^15^. In the present study, of the participants, 25.6% performed BSE, 34.5% underwent mammography, 40.7% planned to undergo mammography (within next 1–5 years), which is consistent with the rates in the literature.

Healthcare professionals, particularly those who regularly care for women, are an important source of breast cancer information for women^6^. Cross-sectional studies conducted to determine nurses’ Knowledge Attitude and Practice (KAP) of breast cancer in Turkey^21^ and Jordan^22^, have demonstrated that nurses’ breast cancer-related KAP range from relatively low to 100%.Their attitudes towards and knowledge of breast cancer screening methods and practice varied greatly.

Of studies conducted in countries other than the aforementioned ones, while some indicated that nurses’ knowledge of risk factors for breast cancer was low^21,22^, others indicated that their level of knowledge was high^23^. Therefore, considering the presence of nurses with low levels of knowledge on the risk factors for breast cancer, more efforts should be made to improve their knowledge. For instance, providing short-term on-site courses especially for nurses who do not work in maternity units/wards can update their knowledge^24^. It has been found that the level of practicing breast cancer screening among nurses is low. Therefore, conducting training programs aimed at updating nurses’ knowledge of risk factors for breast cancer and encouraging them to practice breast cancer screening could help them form a habit of performing such practices, which, though indirectly, increases the general population’s breast cancer-related knowledge and practices^25^. In the present study, 63.7% of the participants were advised to undergo mammography by midwives. That the provision of such advice is mostly performed by midwives is noteworthy.

Its high morbidity and mortality make cancer a crucial public health problem. Ensuring a safer sex life and early diagnosis greatly contributes to the prevention of cervical cancer^26^. Thanks to Pap testing, cervical cancer can be detected early and thus the chance of recovery is quite high. The mean age of patients when they are diagnosed with cervical cancer is 51 years. There are two periods in life when cervical cancer rates peak: between the ages of 35 and 59, and 60 and 64 years^25^. Among the factors affecting women’s undergoing Pap tests are their views on gynecological examination, sociodemographic characteristics, knowledge of Pap test and perception of cervical cancer risk^26^. However, in the Smith et al.^27^ study, some other factors such as women’s economic conditions, presence of health insurance, low personality characteristics and negative feelings were found to affect their Pap testing behaviors. Studies conducted with women of Latin origin demonstrated that women’s cultural and economic statuses also affected their decision of having a Pap test^28^. In the Pinar et al.^29^ study, of the participating nurses, 73.6 % did not have regular gynecological examinations, and 70 % did not have a Pap test previously. In the Wong et al.^30^ study, the participating women either lacked knowledge of cervical cancer or were unaware of cervical cancer. The women who participated in the study by Duran^31^ were not aware of the prevention and early diagnosis of cervical cancer and had almost no fear of it. Uysal and Birsel^32^ conducted a survey of women’s levels of knowledge of cervical cancer and their attitudes towards Pap test and determined that one-third of the participating women underwent Pap test at least once and that there was a close correlation between their knowledge of risk factors for cervical cancer and having a Pap test. In the present study, of the participants, 38.5% had a Pap test and 38.7% planned to undergo a Pap test (within next 1–5 years), which is consistent with the rates in the literature. Women obtain information about Pap tests mostly from medical staff. This is probably because they trust the advice given by healthcare staff and do not hesitate to fulfill it. In the McMullin et al.^28^, the participants started to have a Pap test more regularly after they were informed about it by medical staff. Another study that drew attention to the importance of getting information from medical staff was performed by Wong et al.^30^. In the present study, 45.9% of the participants lacked knowledge of the Pap test. On the other hand, it is noteworthy that advice on the Pap test was from midwives for 70.2% of the participants.

## CONCLUSIONS

While one-fourth of the participants stated that they performed BSE and underwent mammography at least once, more than half reported that they did not have a Pap test. Of the factors affecting their not performing BSE and not undergoing mammography and Pap test, the most common was their lack of knowledge about the procedure, which suggests that the public should be educated on BSE, mammography and Pap test, and that primary responsibility of providing education lies with healthcare professionals especially midwives. In preventive healthcare services, periodic health examinations and screenings for the most common types of women cancers should be increased, midwives should give education and counseling, and the importance of practices aimed at raising social awareness should be emphasized.

## References

[1] International Agency for Research on Cancer (2014). World Cancer Report 2014.

[2] T.C. Sağlık Bakanlığı Sağlık İstatistikleri Yıllığı: 2010 [ Health Statistics Yearbook: 2010].

[3] Wadler BM, Judge CM, Prout M, Allen JD, Geller AC (2011). Improving breast Cancer control via the use of community health Workers in South Africa: a critical review. J Oncol.

[4] Anderson BO, Braun S, Lim S, Smith RA, Taplin S, Thomas DB (2003). Early detection of breast cancer in countries with limited resources. Breast J.

[5] Ersumo T (2006). Breast cancer in an Ethiopian Population, Addis Ababa. East and Central African Journal of Surgery.

[6] Thomas DB, Gao DL, Ray RM (2002). Randomized trial of breast self-examination in shanghai: final results. J Natl Cancer Inst.

[7] Wong LP, Wong YL, Low WY, Khoo EM, Shuib R (2008). Cervical Cancer Screening Attitudes and Beliefs of Malaysian Women Who Have Never Had a Pap Smear: A Qualitative Study.

[8] World Health Organization Cancer.

[9] Cancer Early Diagnosis, Screening and Training Center http://www.ketem.org/.

[10] International Agency for Research on Cancer (2008). World Cancer Report 2008.

[11] World Health Organization World cancer report 2008.

[12] Sankaranarayanan R, Ramadas K, Thara S (2011). Clinical Breast Examination: Preliminary Results from a Cluster Randomized Controlled Trial in India. J Natl Cancer Inst.

[13] Çelik OG, Malak TA, Öztürk Z, Yılmaz D (2009). Examination of postmenopausal women self breast examination, mammography and pap smear. Anatol J Clin Investig.

[14] Akinola R, Wright K, Osunfidiya O, Orogbemi O, Akinola O (2011). Mammography and mammographic screening: are female patients at a teaching hospital in Lagos, Nigeria, aware of these procedures?. Diagn Interv Radiol.

[15] Duman NB, Algier L, Pınar G (2013). Health beliefs of the female academicians about breast cancer and screening tests and the affecting factors. International Journal of Hematology and Oncology.

[16] Durvasula RS, Regan PC, Ureno O, Howell L (2006). Frequency of cervical and breast cancer screening rates in a multi-ethnic female college sample. Psychol Rep.

[17] Duman NB, Yılmazel G, Pınar G, Büyükgönenç L (2015). The risk level of breast cancer and breast cancer awareness among the turkısh women aged 65 years and older. International Journal of Hematology and Oncology.

[18] Lostao L, Joiner T, Pettit JW, Chorot P, Sandin B (2001). Health beliefs and illness attitudes as predictors of breast cancer screening attendance. Eur J Public Health.

[19] Dewal L (2006). Testicular and breast self-examination knowledge and practices of certified athletic trainers and the secondary prevention of such cancers in intercollegiate student-athletes. Am J Health Stud.

[20] Altunkan H, Akın B, Ege E (2008). 20-60 Yaş arası kadınların kendi kendine meme muayenesi uygulama davranışları ve farkındalık düzeyleri [Breast self-examination (BSE) application behaviors and awareness levels of women aged 20-60]. Meme Sağlığı Dergisi.

[21] Andsoy II, Gul A (2014). Breast, cervix and colorectal cancer knowledge among nurses in Turkey. Asian Pac J Cancer Prev.

[22] Alkhasawneh IM (2007). Knowledge and practice of breast cancer screening among Jordanian nurses. Oncol Nurs Forum.

[23] Awodele O, Adeyomoye AA, Oreagba IA (2009). Knowledge, attitude and practice of breast cancer screening among nurses in Lagos University teaching hospital, Lagos Nigeria. Nigerian Quarterly Journal of Hospital Medicine.

[24] Andegiorgish AK, Kidane EA, Gebrezgi MT (2018). Knowledge, attitude, and practice of breast Cancer among nurses in hospitals in Asmara, Eritrea. BMC Nurs.

[25] Kaya M (2009). Cervical cancer with public health approach.

[26] Ak M, Canbal M, Turan S, Gürbüz N (2010). Aile hekimliği polikliniğine başvuran kadınlarda pap smear testinin farkındalığının değerlendirilmesi. Konuralp Tıp Dergisi.

[27] Smith M, French L, Barry CH (2003). Periodic abstinence from pap (PAP) smear study: women's perceptions of pap smear screening. Ann Family Med.

[28] McMullin MJ, De Alba I, Chavez LR, Hubbell FA (2005). Influence of beliefs about cervical cancer etiology on pap smear use among latina immigrants. Ethn Health.

[29] Pınar G, Algıer L, Colak M (2008). Determination of nurses' knowledge about cervical cancer and HPV vaccine. Turkish Journal of Gynecological Oncology.

[30] Wong LP, Wong YL, Low WY, Khoo EM, Shuib R (2009). Knowledge and awareness of cervical cancer and screening among Malaysian women who have never had a Pap smear: a qualitative study. Singapore Med J.

[31] Duran TE (2011). Examination with the health belief model of women's attitudes to cervical cancer and early diagnosis in Turkey: a qualitative study. Asian Pac J Cancer Prev.

[32] Uysal A, Birsel A (2009). Knowledge about cervical cancer risk factors and pap testing behaviour among Turkish women. Asian Pac J Cancer Prev.

